# Therapeutic potential of bleomycin plus suicide or interferon-β gene transfer combination for spontaneous feline and canine melanoma

**DOI:** 10.18632/oncoscience.387

**Published:** 2017-12-28

**Authors:** Lucrecia Agnetti, Chiara Fondello, Marcela S. Villaverde, Gerardo C. Glikin, Liliana M. E. Finocchiaro

**Affiliations:** ^1^ Unidad de Transferencia Genética Instituto de Oncología “Ángel H. Roffo” Universidad de Buenos Aires, Buenos Aires, Argentina

**Keywords:** melanoma, HSV-thymidine kinase, interferon-β, bleomycin, spheroids

## Abstract

We originated and characterized melanoma cell lines derived from tumors of two feline and two canine veterinary patients. These lines reestablished the morphology, physiology and cell heterogeneity of their respective parental tumors. We evaluated the cytotoxicity of bleomycin (BLM) alone, or combined with interferon-β (IFN-β) or HSVtk/GCV suicide gene (SG) lipofection on these cells. Although the four animals presented stage III disease (WHO system), SG treated feline tumors displayed stable disease in vivo, while the canine ones exhibited partial response. Their derived cell lines reflected this behavior. Feline were significantly more sensitive than canine cells to IFN-β gene transfer. BLM improved the antitumor effects of both genes. The higher levels of reactive oxygen species (ROS) significantly correlated with membrane and DNA damages, emphasizing ROS intervention in apoptotic and necrotic cell death. After 3 days of BLM alone or combined with gene treatments, the colony forming capacity of two canine and one feline treatments survivor cells almost disappeared. Taken together, these results suggest that the treatments eradicated tumor initiating cells and support the clinical potential of the tested combinations.

## INTRODUCTION

Some types of human cancer are very similar to the corresponding disease in companion animals. The auspicious results, derived from immunogene therapy studies carried out in companion animals, warrant their usage in veterinary clinical oncology [1, 2]. Progress made with veterinary cancer bearing patients can significantly speed up translational research and benefit both veterinary and human patients.

Malignant melanoma (MM) is rare in cats while is one of the most common tumors in dogs [1-6]. Being clinically similar, both diseases are chemo and radioresistant and share similar phenotypes [3-6]. Locally growing at first, feline and canine MM eventually will invade the lymph nodes and spread to other areas of the body. The internal organs most commonly affected are the lungs, but it can spread to any part of the body [3-6]. These extremely aggressive spontaneous tumors, frequently resistant to current therapies, urgently need new therapeutic strategies.

Intratumor non-viral suicide gene (SG) therapy with thymidine kinase from the herpes simplex virus (HSVtk), in combination with the pro-drug ganciclovir (GCV), has been extensively and successfully used for treating canine malignant diseases [1, 2, 5, 6]. On the other hand, Interferon-β (IFNβ) has antitumor effects against melanoma, and generally is more potent than IFNα [6, 7]. Even though clinically effective, the treatment with recombinant hIFNα/β protein is associated with substantial systemic toxicity that worsens the patient's quality of life and often interferes with the therapy completion [8]. However, the exogenously added recombinant IFNβ protein (rIFNβ) can be successfully replaced by the transfer of the corresponding gene *in vitro* [7]. Local non-viral delivery of the gene encoding this cytokine provides a slow release transgenic system limited to a small area, avoiding the adverse events associated to the injection of high doses of recombinant interferon protein while keeping its therapeutic potential [6]. In addition, lipoplexes can facilitate the delivery of bleomycin (BLM) into melanoma cells via endocytosis [9]. This antineoplastic agent enhances the cytotoxic effects of both SG and IFNβ gene expression on human melanoma and sarcoma cells [10].

Generally, these studies use established tumor cell lines that were kept in culture for many generations, making them very different from the original tumors. Conversely, companion animals' primary melanoma cell lines, could offer alternative promising models for optimizing and predicting the *in vivo* response of their respective tumors to therapeutic strategies [11]. Besides, few stable feline and canine melanoma cell lines are currently available. Thus, we established and characterized four melanoma cell lines derived from surgically excised canine and feline melanoma tumors. On these lines, we explored the therapeutic potential of the combination of BLM with IFNβ gene and SG lipofection.

## RESULTS

### Melanoma cell lines were derived from highly malignant in vivo tumors

To evaluate potential *in vivo* responses of individual spontaneous feline and canine melanomas to our treatments, we established and characterized four melanoma cell lines, two feline (*Dc* and *Rn*) and two canine (*Bsk* and *Rk*). They derived from surgically excised feline oral (*Dc*) and abdominal (*Rn*) and canine oral mucosal melanoma (*Bsk* and *Rk*) advanced tumors of veterinary patients.

Dc feline patient was bearing a highly malignant gingival mass of 3.1 cm mean diameter expanding to the ipsilateral lymph node (mean diameter 2.2 cm).

Rn feline patient: was carrying two large lesions (mean diameter: 7.2 cm and 5.9 cm respectively) of an abdominal highly advanced melanoma with metastatic spread all over the abdomen.

Bsk canine patient: was bearing a recurrent oral tumor progressing on the right maxilla of 3.3 cm mean diameter spread to the ipsilateral lymph node (mean diameter 5.7 cm).

Rk canine patient: was bearing a primary melanoma on the left maxilla of 4.7 cm mean diameter with an osteolytic metastatic spread.

### Melanoma cell lines displayed considerable heterogeneity

Melanoma cell lines derived from feline and canine tumors of our veterinary patients seem to be able to re- establish the morphologic and physiologic phenotype of their respective in vivo tumors. Compatible with the clinical diversity of this disease, feline and canine melanoma cells displayed high heterogeneity related to the high variability of cell populations [5, 6, 12, 13].

Feline cell lines (*Dc*,*Rn*) displaying significantly shorter duplication times (DT≅18-20 h), were faster growing than the canine ones (*Bsk*,*Rk*) with DT≅28-30 h. However, the four cell lines exhibited similar proliferation index (PIx) as the proportion of cells in S, G /M and hyperdiploid phases (Table 1).

**Table 1:** Characteristics of cultured melanoma cells

Cell line	*Dc*	*Rn*	*Bsk*	*Rk*
**Origin**	oral	abdominal	oral	oral
**Cell morphology**	epithelioid	fibroblastic	fibroblastic	fibroblastic
**Spheroid morphology**	loose (multiple)	compact	loose	loose
**Lipofection rate (%) [n]**	3.01 ± 1.61 [8]	1.80 ± 0.71 [12]	26.54 ± 7.23 [15]**	16.81 ± 6.91 [12]*
**Duplication time (h) [n]**	18.08 ± 1.90 [3]	19.89 ± 2.49 [4]	28.87 ± 2.36 [4]*	29.93 ± 1.09 [3]*
**Proliferation Index (%) [n]**	41.18 ± 1.03 [4]	47.35 ± 2.00 [4]	48.07 ± 4.19 [3]	39.12 ± 5.28 [6]
**ROS (geometric mean) [n]**	21.47 ± 4.02 [4]	15.41 ± 2.00 [4]	36.73 ± 3.50 [4]**	51.26 ± 2.98 [3]***
**Cell granularity (mean) [n]**	999.85 ± 86.06 [7]***	1720.73 ± 109.18 [6]	913.07 ± 158.93 [7]**	996.06 ± 55.70 [6]*
**Cell size (mean) [n]**	2024.80 ± 204.89 [3]*	1564.45 ± 141.43 [3]	1875.11 ± 138.17 [3]*	1941.58 ± 116.77 [3]*
**Clonogenic capacity (% seeded cells) [n]**	9.14 ± 2.35 [7]***	33.11 ± 1.66 [3]	11.68 ± 1.95 [6]***	3.90 ± 0.50 [3]***
**Colonies in soft agar (% seeded cells) [n]**	13.08 ± 1.17**	22.83 ± 1.86	9.16 ± 1.03***	5.37 ± 0.53***
**Melanosphere forming capacity (%)**	n.d.	11.18 ± 5.53 [3]	6.715 ± 0.12 [2]	n.d.

Time course of growth was determined by trypan blue exclusion cell counting and lipofection efficiency was measured as blue X-Gal stained cells as described in Materials and methods. Proliferation index (PIx) of untreated cells was determined by evaluating the percentage of cells in the S, G2/M and hyperdiploid phases. ROS: Reactive oxygen species. n.d.: not determined. * vs Rn respective value. One symbol: p<0.05, two symbols: p<0.01, three symbols: p<0.001.

In agreement with the highly malignant and invasive Rn tumor, *Rn* derived cell line also displayed a more aggressive phenotype by forming respectively 2-, 2- and 4-fold more colonies in soft agar; and 3-, 3- and 7- fold more adherent colonies than *Dc, Bsk* and *Rk*. Accordingly, *Rn* cell line displayed the greatest proportion of cells with lower basal ROS levels, lower size and higher complexity (Table 1). All these characteristics have been associated with a pluripotent/stem cell phenotype [14-18].

### Feline and canine melanoma cells were able to form colonies and melanospheres

The four melanoma cell lines, when seeded at low density, were able to grow as colonies, either in suspension (soft agar) or under adherent conditions.

**Figure 1:**
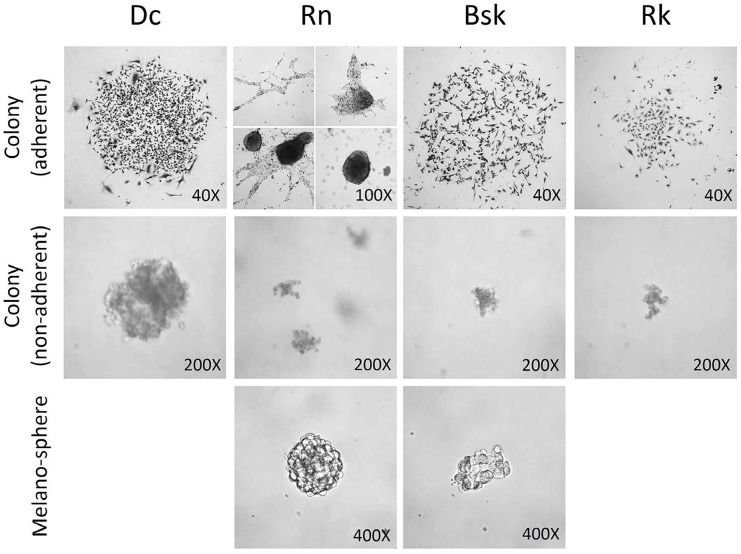
Colonies morphology under adherent and non-adherent (in soft agar) conditions and melanosphere morphology Colonies and melanospheres growing under adherent or non adherent conditions, as described in Materials and Methods, were photographed using a Nikon eclipse TE2000-S inverted phase contrast microscope.

Under non-adherent conditions, the four cell lines formed colonies of different morphology when seeded at the same concentration. *Dc* produced the biggest spherical colonies, while *Rn, Bsk* and *Rk* tended to form small irregular aggregates (Fig.1).

On the other hand, the shape of the colonies formed under adherent conditions was very different from those in soft agar. *Dc, Bsk* and *Rk* tended to form spherical aggregates of looser structure. *Rk* ones adopted a smaller and lax structure. Consistent with the high heterogeneity of cell populations, *Rn* tended to form both elongated aggregates and dense spherical colonies displaying a spreading pattern. After reaching a definite size, *Rn* colonies spontaneously became dense spherical masses that easily detached and persisted at the supernatant of the well plate (Fig.1).

Moreover, feline *Rn* and canine *Bsk* melanoma cells were able to form round and compact melanospheres when seeded under non-adherent and serum-free conditions (Fig.1).

### Specific markers evidenced the invasive and proliferative status of feline and canine melanoma cells

**Figure 2:**
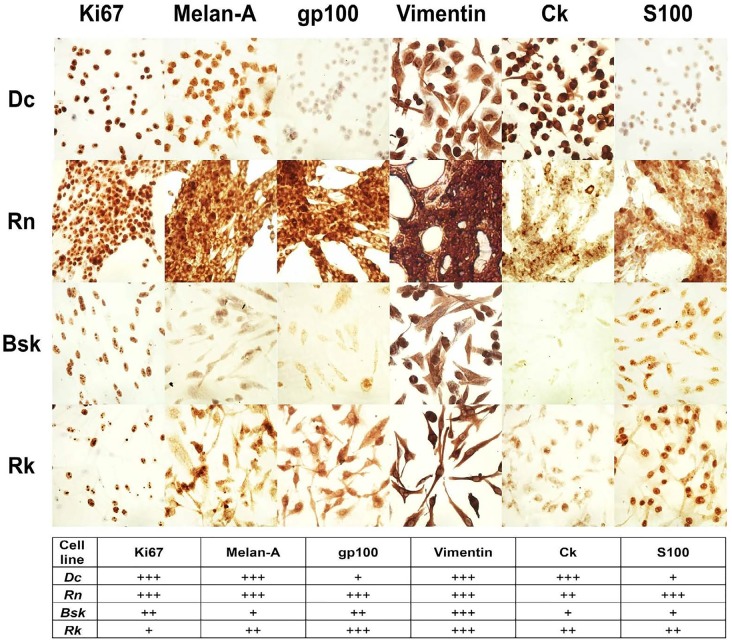
Detection of melanoma specific antigenic markers by immunocitochemistry Cells growing for 2 days onto glass slides were fixed and stained with specific antibodies against ki67, melan-A, gp100, vimentin, cyitokeratin (Ck) and S100A9 as described in Materials and methods and photographed (400X). The number of + symbols represents a semi-quantitative estimation of the relative staining of each individual tumor marker among the four cell lines.

Consistent with its faster growing, *Dc* and *Rn* nuclei were highly positive for the specific proliferation marker Ki67 (Fig. 2). The expression of this a nuclear antigen, indicator of proliferating cells [19], was moderate in *Bsk* and low in *Rk*.

In addition, Melan A and gp100, two specific and sensitive melanoma antigens associated with cell proliferation programs [20], were very elevated in *Rn* cell line. Melan A (expressed in pigmented cells) was also high in *Dc*, moderate in *Rk* and low in *Bsk*. Gp100 (expressed in activated melanocytes) was high in *Rk*, moderate in Bsk and low in *Dc*.

In agreement with its fibroblastic phenotypes (Table 1), Rn, Bk and Rk melanoma cell lines, were positive for the mesenchymal marker vimentin (Fig. 2). Even *Dc*, displaying a more epithelioid morphology showed a vast subpopulation of vimentin expressing cells. It is worth to note that, the four vimentin positive cell lines, also co-expressed cytokeratin (CK, a keratinocyte specific marker), confirming their invasive and metastatic behavior [21].

On the other hand, S100A9, a member of S100 family, was high in *Rn*, moderate in *Rk* and low in *Dc* and *Bsk* (Fig. 2). S100A9 (myeloid-related protein 14), implicated in the abnormal differentiation of myeloid cells in the cancer stroma, contributes to create an immunosuppressive microenvironment that inhibits the generation of a protective cellular immune response by the tumor-bearing host [22]. Furthermore, only *Rn* expressed the lysosome-associated glycoprotein CD68 (data not shown).

Beyond depicting their proliferative and invasive status, the morphologic analysis and the positive staining for most of the assayed markers confirmed the previous histopathological diagnosis of melanoma.

### Bleomycin enhanced the cytotoxic effects of both suicide and IFNβ gene lipofection on melanoma cells growing as monolayers and spheroids

The development of new combinations of treatment strategies could extensively target different cancer cell subpopulations overcoming treatment resistance. In a previous work, we found that, the *in vitro* response of tumor derived spheroids (sph) correlated with the clinical outcome of the suicide gene treatment observed on canine melanoma patients *in vivo* [11]. Here, we explored if both, species-specific feline/canine interferon-β (f/cIFNβ) gene and herpes simplex thymidine kinase/ganciclovir (HSVtk/GCV) suicide gene (SG) therapy could be successfully combined with bleomycin (BLM) for treating melanoma derived cell lines growing as monolayers (mnl) or sph. We estimated the BLM effect at 3 μg/ml and SG cytotoxicity at the pharmacologically relevant 5 μg/ml ganciclovir (GCV) concentration, similar to the intratumor standard dose for our feline and canine patients [5-7].

**Figure 3:**
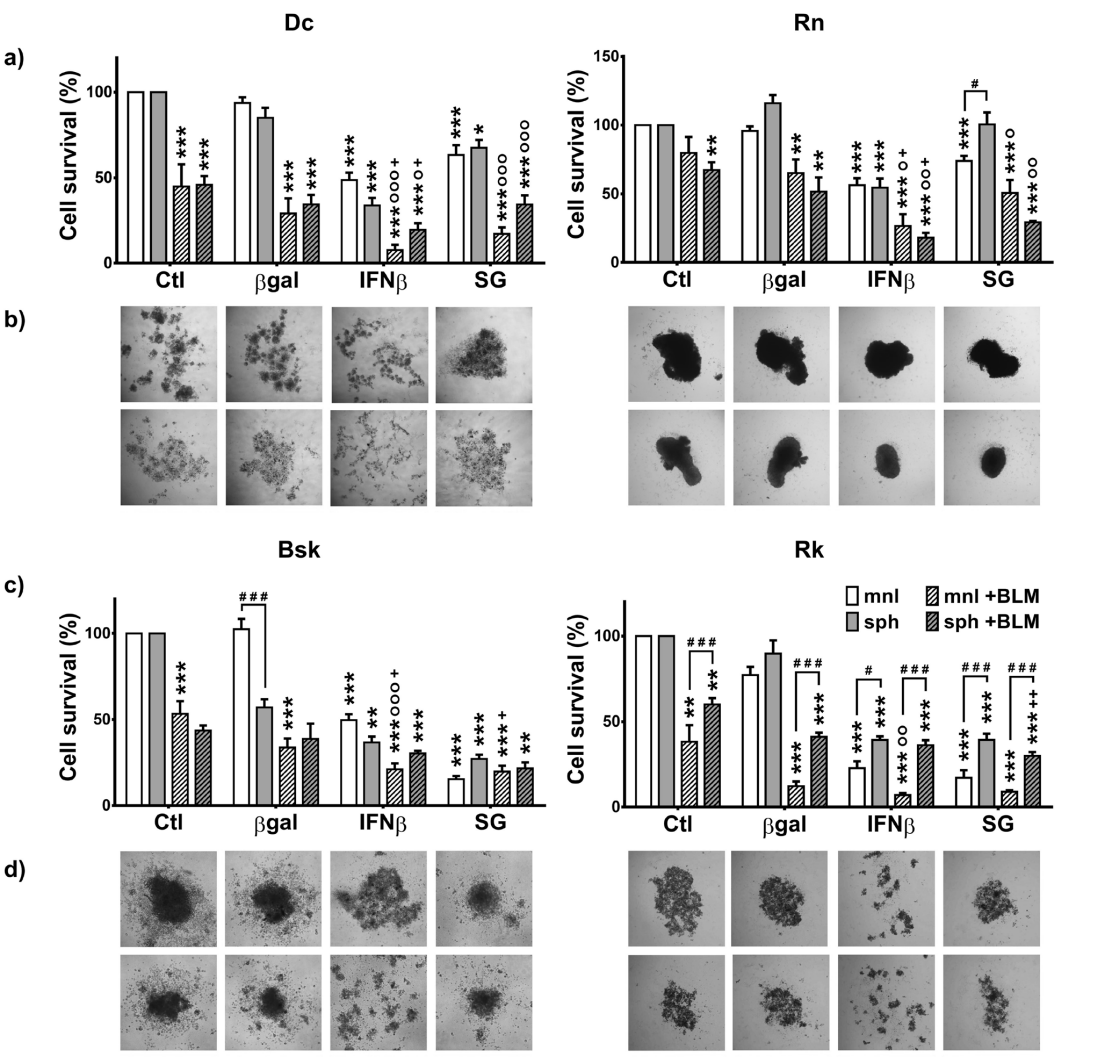
Effect of bleomycin (BLM) on the sensitivity of melanoma cells monolayers and spheroids to interferon-β (IFNβ) gene and suicide gene (SG) system (HSVtk/GCV) as described in Materials and methods (a,c) The results represent means ± s.e.m. of n ≥ 4 (*Rn* and *Bsk*), n ≥ 6 (*Rk*) and n ≥ 8 (*Dc*) independent experiments. In this case each value was relative to the respective control condition: monolayers (mnl) or spheroids (sph). * vs the respective βgal; o BLM vs their respective value without BLM; + BLM/genes combined treatments vs their respective ctl+BLM; # sph vs their respective value in mnl. One symbol: p<0.05, two symbols: p<0.01, three symbols: p<0.001. (b,d) Images represent individual melanoma spheroids treated as described in the bar plots. Spheroids growing in suspension in 96-well plates for 10 days were photographed using an inverted phase contrast microscope (100X).

With the exception of SG in *Rn* growing as sph, the tested cell lines were sensitive to both, IFNβ and SG lipofection in both spatial configurations (Fig. 3). Canine cell lines (*Bsk*, *Rk*), with higher lipofection efficiency (≅16-26%), were more sensitive to SG system. Conversely, feline cell lines (*Dc*, *Rn*), presenting very low lipofection efficiency (≅2-3%), were more sensitive to IFNβ gene in both spatial configurations (Table 1, Fig. 3). Surprisingly, Rn cell line with fair lipofection efficiency (≅2%) was sensitive, not only to fIFNβ in both spatial configurations, but also to SG gene when growing as mnl.

All cell lines were sensitive to BLM alone: *Dc* and *Rk* (mnl and sph), *Bsk* (mnl) and *Rn* (sph). However, unspecific lipofection (βgal) enhanced BLM cytotoxicity in the four lines: *Dc, Bsk* and *Rk* (mnl), and *Rn* (sph). This effect was probably due to lipoplexes-mediated cellular uptake [9]. On the other hand, the combination with BLM enhanced the individual effects of both, fIFNβ and SG gene, in feline lines (*Dc* and *Rn*), and the cIFNβ effects in canine lines (*Bsk*, mnl and sph; and *Rk*, mnl). The highly sensitive canine cell lines, showed equal (mnl) or greater response (sph) to the combination BLM/SG with respect to SG alone (Fig. 3).

*Rk* showed greater response to both, SG and cIFNβ gene, as monolayers than as their respective spheroids and *Rn* did so only in response to SG (Fig. 3). This decreased sensitivity of spheroids would be due to the phenomenon called multicellular resistance (MCR) that reflects the relative intrinsic treatment-resistant phenotype of most solid tumors growing *in vivo* [11].

As observed in Fig. 3b, the microscopic monitoring of treated spheroids paralleled the results obtained in the bar plots.

In agreement with the high heterogeneity of melanoma, the four cell lines were able to grow as multicellular spheroids of different morphologies. The small size and looser structure of *Dc* sph, became more lax after treatments (SG excepted). Conversely, *Rn* cells appeared intimately associated with each other and closely packed. Treatments, specially the combined ones, resulted in a significant reduction of spheroids volume, while maintained their degree of compactness (Fig. 3b).

On the other hand, *Bsk* tended to form spherical aggregates displaying a spread pattern, while *Rk* formed intermediate cell clusters. In both canine cell lines, cIFNβ gene lipofection alone and combined with BLM caused a morphological change consistent of multiple small aggregates of cells loosely associated with each other. The rest of the treatments decreased the spheroid size while maintained their morphology.

### *In vitro* suicide gene sensitivity correlated with *in vivo* tumor response of feline and canine melanoma patients

Although the four patients presented stage III disease (tumor > 4 cm and/or positive nodes), as defined by WHO staging system, the variability of responses between individual patients to SG treatment, was compatible with the clinical diversity of this disease.

During our veterinary clinical study, Bsk and Rk patients displayed a partial response to SG [5], while Dc presented stable disease during the 5 weekly suicide gene treatments [Finocchiaro et al., unpublished data]. On the other hand, the enormous and highly invasive Rn tumor showed a fast progression in situ before gene therapy, with both large lesions doubling their volume in only 3 weeks (from 57.6 to 113.3 cm3 and from 47.2 to 86.7 cm3). Tumor growth stopped during the 5 weekly treatments with SG, becoming a stable disease. There was little change in size for the following 10 weeks and after that growth resumed [Finocchiaro et al., unpublished data].

### Chemo-gene treatments increased the fraction of hypodiploid subG_0_ apoptotic-necrotic cells

Cells dying by apoptosis, a regulated cell death, activate endonucleases that cleave DNA in fragments of approximately 180-200bp. Conversely, necrotic cell death occurs drastically, leading to plasma membrane permeabilization and to a rapid non-specific cleavage of DNA. Thus, cells undergoing apoptosis/necrosis (A/N) can be readily identified by flow cytometry as cells with hypodiploid or subG DNA content after propidium iodide staining.

**Figure 4:**
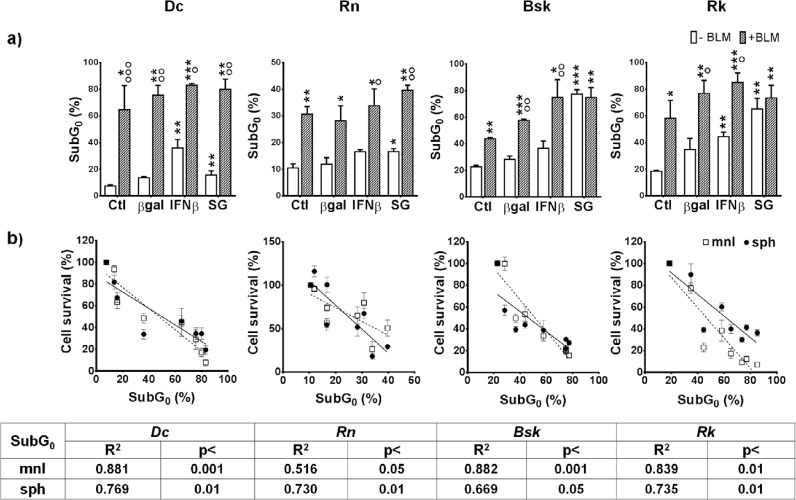
Effects of IFNβ gene and SG (HSVtk/GCV) lipofection in the absence or presence of BLM on apoptotic-necrotic cell death of melanoma cells (a) The fraction of hypodiploid subG apoptotic cells was determined by flow cytometry as described in M&M. The results represent means ± s.e.m. of n ≥ 3 independent experiments. * vs the respective β-galactosidase gene (βgal); o BLM vs their respective value without BLM; + BLM/genes combined treatments vs their respective ctl+BLM; + BLM/genes combined treatments vs their respective βgal+BLM. One symbol: p<0.05, two symbols: p<0.01, three symbols: p<0.001. (b) Correlations between hypodiploid subG0 apoptotic cells and cells survival to all treatments in mnl (□) or sph (•) were determined by Pearson test with GraphPad Prism program.

The levels of DNA breakup observed in the cells treated with βgal lipofection alone were not significantly different from those observed in unlipofected control cells. SG produced a significant increase of cells accumulated in the hypodiploid subG A/N region in the four cell lines. IFNβ gene lipofection did so in *Dc* and *Rk*. Interestingly, BLM alone or combined with gene treatments, increased even more this fraction of cells in the four cell lines (Fig. 4a).

On the other hand, the four cell lines exhibited an inverse correlation between the fraction of hypodiploid subG_0_ A/N cells and cell survival to all the treatments in both spatial configurations (Fig. 4b).

### The treatments increased the fraction of necrotic cells

In contrast to the apoptotic pathway, necrosis has obvious implications for the *in vivo* success of treatments. The intracellular content leaking to the extracellular space through damaged plasma membrane may induce the development of *in vivo* strong immune response that could provide additional antitumor activity. In order to quantify this process, we measured propidium Iodide (PI) uptake by unfixed necrotic cells with permeable plasma membranes.

**Figure 5:**
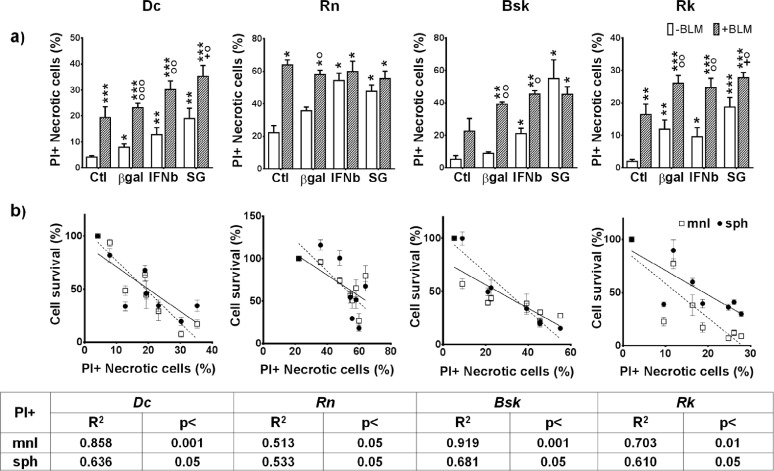
Effects of IFNβ gene and SG (HSVtk/GCV) lipofection in the absence or presence of BLM on necrotic cell death of melanoma cells (a) The fraction of necrotic propidium iodide (PI) stained cells was determined by flow cytometry as described in Materials and methods. The results represent means ± s.e.m. of n > 3 independent experiments. * vs the respective β-galactosidase gene (βgal); O BLM vs their respective value without BLM; + BLM/genes combined treatments vs their respective ctl+BLM; One symbol: p < 0.05, two symbols: p < 0.01, three symbols: p<0.001. (b) Correlations between necrotic propiduim iodide (PI) stained cells and cells survival to all treatments in mnl (□) or sph (•) were determined by Pearson test with GraphPad Prism program.

Three days after IFNβ gene and SG lipofection the four cell lines significantly increased the necrotic cell death. BLM alone or combined with both genes induced a significant additional increase of necrotic cells (Fig. 5a). On the other hand, the four cell lines exhibited an inverse correlation between the fraction of necrotic cells and cell survival to all the treatments in both spatial configurations (Fig. 5b).

### Chemo-gene treatments increased the subpopulation of cells with high ROS content

Reactive oxygen species (ROS) play a major role as mediators of IFNβ gene cytotoxic effects in human and canine melanoma cell lines [7]. Thus, we explored a possible link between the cytotoxicity of BLM- gene treatments and the increase in intracellular ROS in our new established cell lines.

**Figure 6:**
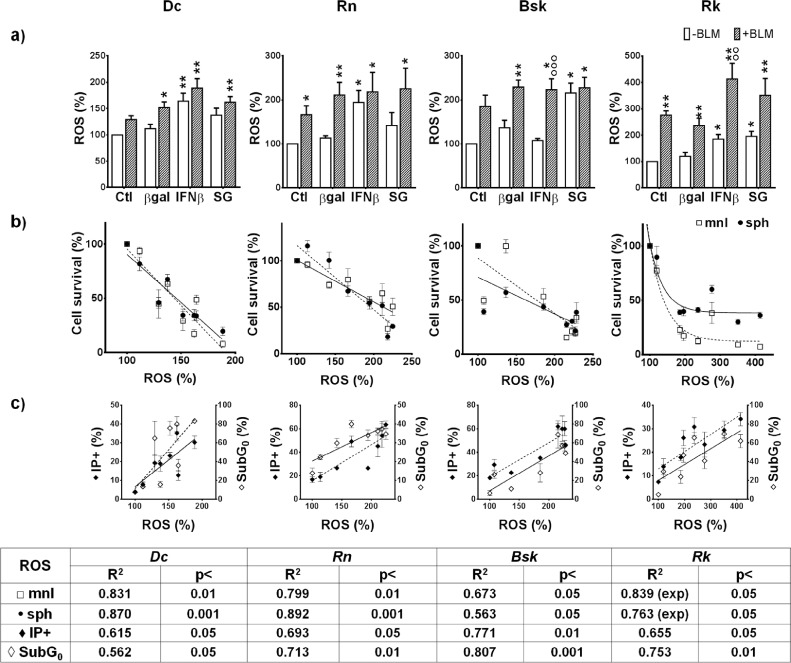
Effects of SG (HSV*tk*/*GCV*) and IFNβ lipofection in the absence or presence of BLM on intracellular reactive oxygen species (ROS) production by melanoma cells Intracellular ROS levels were measured by means of H2DCF-DA probe as described in Materials and methods. (a) The results represent means ± s.e.m. of n ≥ 6 (*Bsk* and *Dc*), and n ≥ 3 (*Rn* and *Rk*) independent experiments. * vs their respective βgal; o BLM vs their respective value without BLM; + BLM/genes combined treatments vs their respective βgal+BLM. One symbol: p<0.05, two symbols: p<0.01, three symbols: p<0.001. Correlations between the fraction of cells with high levels of intracellular *ROS* and (b) cells survival to all treatments in mnl (□) or sph (•) or (c) SubG0- apoptotic cells (◊) and PI+ necrotic cells death (♦) were determined by Pearson test with GraphPad Prism program.

Compared to control cells, we observed that, a subpopulation of cells with high intracellular levels of ROS was significantly increased by IFNβ gene, in feline (*Dc* and *Rn*) and canine (*Rk*) melanoma cells. Conversely, SG induced a significant ROS increase only in canine cell lines (*Bsk*, *Rk*) (Fig. 6a). It is worth to note the high increase of ROS produced by BLM alone or combined with IFNβ gene and SG lipofection in the four cell lines. (Fig. 6a).

The rise in ROS levels correlated with the extent of the cytotoxic response. The four cell lines exhibited an inverse correlation (p<0.05) between intracellular ROS levels and cell survival to the chemo-gene treatments in both spatial configurations (Fig. 6b).

On the other hand, we found a direct correlation between the subpopulation of cells with high intracellular levels of ROS and the fraction of hypodiploid subG_0_A/N cells (Fig.6c), and with the proportion of necrotic cells with PI-positive nuclei (Fig. 6c), in the four cell lines.

### The treatments modified the fraction of cycling cells

In a previous paper we demonstrated that, opposing to the treatment, there is a repopulation (re-growth) mechanism whose strength would be intrinsic of each individual tumor [11].

**Figure 7:**
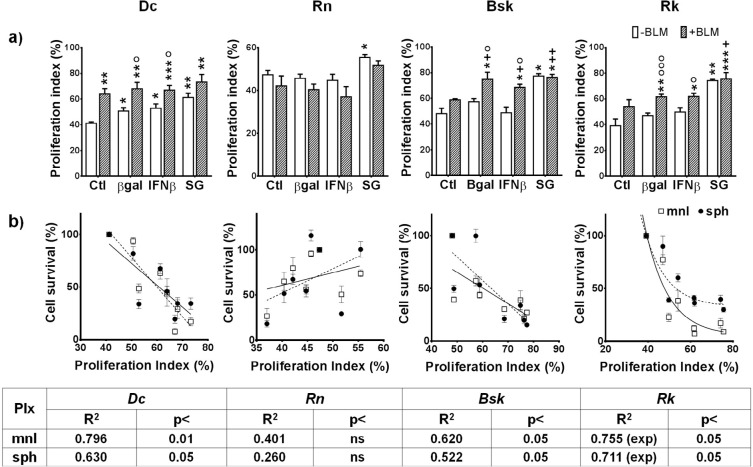
Effects of SG (HSV*tk*/GCV) and IFNβ lipofection in the absence or presence of BLM on the proliferation index (PIx) of melanoma cells (a) Cells growing for 3 days as monolayers were suspended, treated and subjected to flow cytometry analysis as described in Materials and methods. Proliferation index (PIx) was determined by evaluating the percentage of cells in the S, G /M and hyperdiploid phases. The results represent means ± s.e.m. of n ≥ 3 independent experiments. * vs their respective ctl; o BLM vs their respective value without BLM; + BLM/genes combined treatments vs their respective ctl+BLM. (b) Correlations between PIx and survival cells to all treatments in mnl (□) or sph (•) cells were determined by Pearson test with GraphPad Prism program. One symbol: p<0.05, two symbols: p<0.01, three symbols: p<0.001.

In canine cell lines, SG and combined treatments increased the proportion of cells with high proliferation index (PIx) (Fig. 7a). Furthermore, both single and combined treatments enhanced the proliferative phenotype of *Dc* cells. Conversely, only SG enhanced the proliferative fraction of *Rn* feline cell line.

On the other hand, *Dc, Bsk,* and *Rk* cell lines, exhibited an inverse correlation between the fraction of cells with high PIx and the extent of cell survival to treatments in both spatial configurations (Fig. 7b).

### Surviving tumor cells displayed a reduced clonogenic capacity after treatments

**Figure 8:**
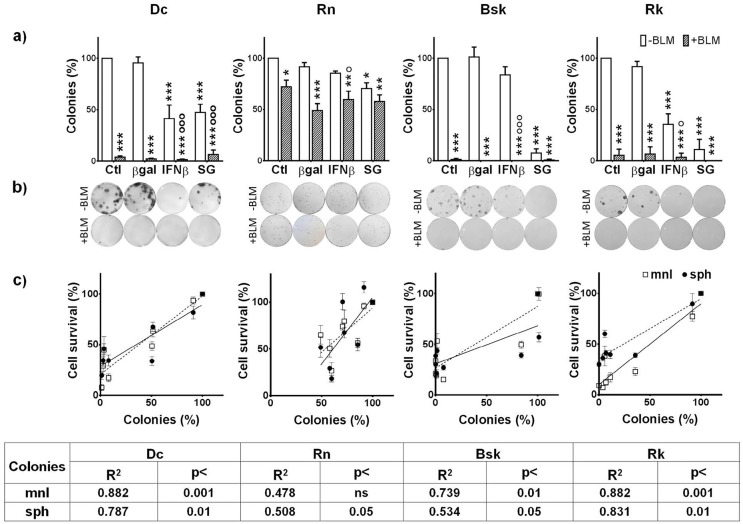
Clonogenic forming capacity of the surviving melanoma cells to SG (HSV*tk*/*GCV*) and IFNβ lipofection in the absence or presence of BLM relative to the number of seeding cells, as described in Materials and methods (a) The results represent means ± s.e.m. of n ≥ 3 independent experiments. * vs their respective βgal; o BLM vs their respective value without BLM; + BLM/genes combined treatments vs their respective βgal+BLM. One symbol: p<0.05, two symbols: p<0.01, three symbols: p<0.001. (b) Images of a representative experiment. c) Correlations between surviving cells in mnl (□) or sph (•) to all treatments and its clonogenic forming capacity as determined by Pearson test with GraphPad Prism program.

Due to the re-growth resistance effect found after an early fast destruction of tumor cells both *in vivo* and *in vitro*, the long term outcomes of the treatment often differ from short term cytotoxic effects. Clonogenic survival studies are useful for to evaluate these long term consequences and confirmed that the combination was more effective than the individual treatments (Fig. 8a,b).

Despite the great colony-forming capacity (CFC) of *Rn* untreated cells (Table 1), surviving cells to both SG and BLM (alone or combined treatments) exhibited a significantly decreased CFC.

It is worth to note that the clonogenic capacity of *Dc, Bsk* and *Rk* surviving cells almost disappeared after treatment with BLM alone or combined with genes (Fig. 8a).

Fig. 8b images represent examples of the clonogenic capacity of the surviving cells to treatments described in the bars plots. *Dc, Bsk* and *Rk* cell lines formed growing colonies that were visible in plain sight 10-12 days (*Dc* and *Bsk*) or about 20 days (*Rk*) after seeding. *Rn* colonies were not able to reach the same size of the rest at any time, as they spontaneously became dense spherical colonies that easily detached and persisted at the supernatant of the well plate.

On the other hand, there was a direct correlation between the fraction of surviving cells after any treatment and their CFC in *Dc*, *Bsk* and *Rk* cell lines (Fig. 8c). The higher CFC of *Rn* cells did not correlate with the extent of the cytotoxic responses.

## DISCUSSION

Tumors are formed from distinct cell populations. This heterogeneity of tumor structure leads to the occurrence of sensitivity and resistance to applied treatment. One of the critical issues in designing efficient therapies is to understand the composition of heterogeneous melanoma tumors to target treatment resistant cell subpopulations. Here, we established and characterized two feline (*Dc* and *Rn*) and two canine (*Bsk* and *Rk*) melanoma cell lines from veterinary patients. These cell lines, arose from highly proliferative (*Rn>Bsk>Dc>Rk*) and highly invasive (*Rn>Bsk>Dc>Rk*) advanced tumors. Although the four patients presented stage III disease (tumor > 4 cm and/or positive nodes), as defined by WHO staging system, feline *in vivo* tumors (*Dc, Rn*) displayed stable disease (reduction or increase in tumor size <50%, data not shown), while canine ones *(Bsk, Rk*) exhibited partial response (reduction of tumor size > 50%) to SG treatment [5]. This behavior was reflected by their derived melanoma cell lines confirming the behavioral similarity between *in vivo* tumors and their *in vitro* models (Fig. 3).

Even though it was reported that the aggressiveness of human melanoma is promoted by aneuploidy-driven gene expression deregulation [23], ploidy analysis of the four cell lines tested in this work evidenced that they are euploid (*2n*, data not shown). On the other hand in a veterinary setting, survival times in dogs and cats with melanocytic tumors, was not correlated with modifications of DNA content or changes in nuclear morphometry of tumor cells [24]. Therefore the basis for the correlations between *in vivo* and *in vitro* tumor cells behavior should be located elsewhere.

Feline cell lines (*Dc*, *Rn*), were more sensitive to IFNβ gene in both spatial configurations. BLM improved the antitumor effects of both genes, suggesting a useful interaction between them involving powerful cell death mediators. Reactive oxygen species (ROS), triggered by treatments, would be among the main molecules involved in the process [7]. These data, together with the high number of cells with DNA fragmentation and plasma membrane disruption, strongly emphasize the role of ROS as mediators of cell death mostly through apoptotic and necrotic events (Figs. 3-6). Probably BLM, that also increases pro-oxidant species, can complement the antitumor efficacy of the SG and IFNβ gene and clearly causes potentiated effects.

According to the invasive behavior of their respective tumors, the four cell lines were positive for the mesenchymal marker vimentin (Fig. 2). These data suggest that, at the time of tumor resection, a vast subgroup of tumor cells of the total population, had already suffered the epithelial to mesenchymal transition (EMT), increasing their metastatic potential. However, to colonize the metastatic site, cells may reverse in part the EMT by re-expressing basal-like phenotype-associated proteins as cytokeratin (CK) [25]. These vimentin-positive cells also co-expressed CK, confirming their invasive and metastatic behavior [21].

As suggested by Ki67, Melan A and gp100 melanoma antigen expression (Fig. 2), the four cell lines exhibited a proliferative behavior [19]. *Bsk, Dc* and *Rk* lines responded to our single and combined treatments by enhancing the fraction of cells actively proliferating (with high PIx, Fig. 7) and with elevated levels of intracellular ROS (Fig. 6). It was stated that cells with high intracellular ROS levels (Fig. 6) are actively proliferating and more sensitive to therapy and differentiation [14]. Besides, higher ROS state induces proliferation and differentiation of tumor initiating cells (TICs) [14-16]. Conversely, low ROS phenotype is a common property of TICs, required for the maintenance of their self-renewal capacity, quiescent state, high tumorigenicity and therapy resistance [14-16].

It is remarkable that *Rn* cell line, that exhibited the lowest levels of ROS, was also enriched with a high proportion of cells with both, small size and high complexity (Table 1). All these parameters have been associated to TICs [14-18]. This cell line, sensitive to single and combined treatments (Fig. 3-5), only displayed a further increase in the proportion of cells with high basal proliferation index (PIx) in response to SG treatment (Fig. 7). These data indicate that even within highly proliferative melanoma cells, a subpopulation resides in a slow-cycling state. These slow-cycling cells, characteristics of melanoma TICs, are essential for continuous tumor growth [25-29]. According to the massive and highly invasive Rn tumor, long term repopulation by cell re-growth prevail over an early fast destruction by treatments of Rn melanoma cells both *in vivo* and *in vitro* (Fig. 3). The high Ki67 staining (Fig. 2), the spheroids multicellular resistance to SG (Fig. 3), the significant increase of apoptotic and necrotic death in response to treatments (Fig. 4, 5) and the highest clonogenic forming capacity (Fig. 8) of *Rn* surviving cells support this hypothesis. Clonogenic survival studies are useful for to evaluate long-term outcomes of treatments that often differ from short-term cytotoxic effects. When surviving Rn cells (derived from a highly aggressive and metastatic advanced tumor) were seeded *in vitro* at low density, generated 2-, 2- and 4-fold more colonies in soft agar and 3-, 3- and 7-fold more adherent colonies than the other three cell lines (Table 1 and Fig. 8). In addition, Rn cells showed only a small decrease of CFC of surviving cells to SG and BLM (alone or combined with genes) (Fig. 8).

All these data suggest that tumor initiation was not necessarily restricted to a minor population of *Rn* melanoma cells. This cell line may be enriched in both, treatments-sensitive cells and TICs. Thus, treatments only affect a small fraction of *Rn* TICs, by promoting self- renewal or proliferation. Conversely, the higher fraction of this particular set remained quiescent, and then, protected from a harsh environment that induce its exhaustion [25-29]. Possibly, in the other three cell lines the constant cycling imposed by BLM and the sustained expression of IFNβ and SG (Fig. 6), leads to TICs exhaustion [26, 27]. This was in fact observed in Fig. 8, where the CFC of surviving cells almost disappeared after treatment with BLM alone or combined with genes. The high correlation between the fraction of *Dc, Bsk* and *Rk* cells surviving to treatments with their CFC (Fig. 8) suggested that, our chemo-gene treatments, reduced the melanoma TICs compartment in the three lines by promoting long-term TICs self-renewal, repopulating activity and differentiation.

In our spontaneous canine melanoma clinical trials [5], repeated *in vivo* intra tumor administrations of lipid- complexed SG plus GCV yielded 62% of *in vivo* objective responses with 30% complete responses. Since TICs are responsible of tumor recurrence and metastatic disease [28], the high proportion of tumor complete responses and metastasis-free patients suggests that our SG therapy was able to eliminate TICs *in vivo* [5]. In addition, our surgery adjuvant veterinary clinical trials combination of a plasmid DNA vaccine with local SG [5] and SG plus cIFNβ [6] delayed or prevented post-surgical recurrence and distant metastasis while significantly improved disease-free and overall survival of our canine patients. Other combination of BLM (as electrochemotherapy) with a cytokine gene (IL-12) electrotransfer was successfully tested in veterinary patients [30].

The effective clinical outcome of our veterinary clinical trials suggest that *in vivo* continuous and sustained local expression of SG and cIFNβ gene, reduce TICs [5-7], and that such behavior was reflected by their derived melanoma cell lines [11]. In addition, the results presented here suggest that BLM improves the antitumor effects of both genes in both spatial configurations, and produces a moderate (*Rn*) or dramatic reduction (*Dc, Bsk, Rk*) in the CFC of surviving cells in the four melanoma cell lines (Fig. 8a). The previously published clinical data [5, 6] together with the *in vitro* data presented here, suggest that the combination of BLM plus gene treatments could eradicate TICs *in vivo* and strongly supporting the clinic potential of this strategy.

## MATERIALS AND METHODS

### Veterinary patients

Cats and dogs with a confirmed histopathological diagnostic of melanoma were recruited for a study as it was reported [5, 6]. Their owners were notified about the experimental nature of the treatment, and all of them granted written informed consent for treatment [5, 6]. These tumors were staged by a veterinary oncologist according to the WHO staging system of stage III (tumor > 4 cm and/or positive lymph nodes). These spontaneous melanoma veterinary patients received twice a week during 5 weeks, intra- and peri- tumor injection at multiple sites of lipid-complexed plasmid DNA encoding HSVtk (1 to 4 mg DNA co-delivered with 5-20 mg GCV according the tumor size).

### Establishment of cell cultures from feline and canine melanoma tumors

Primary cell lines derived from surgically excised feline oral (*Dc*) and abdominal mucosal melanoma (*Rn*) and canine oral (*Bsk* and *Rk*) melanomas were obtained by mechanical disruption of tumor fragments in culture medium [7, 9]. Periodically tested for mycoplasma absence, cells were cultured as monolayers and multicellular spheroids as described [7, 9].

For doubling time estimation using GraphPad Prism 6 software (GraphPad Software Inc.), cells were trypsinized and 5x104 cells were plated in duplicate in 6-well plates and cultured in normal conditions. After trypan blue staining, cells were daily counted in a Neubauer chamber.

### Immunocytochemistry

Cells attached onto a glass slide were cultured for 48 h in the above described conditions. Cells were then washed, fixed with ethanol, dried, re-hydrated and incubated separately with the following specific antihuman monoclonal antibodies as described by the manufacturers: (i) from BioGenex: melan A (clone A103), S-100A9 (clone 15E2E2), GP100 (clone HMB45); (ii) from Dako: cytokeratin (clones AE1/AE3), vimentin, CD68 and Ki67. After washing, cells were incubated 2 h at room temperature, with Multi-Link immunoglobulins (BioGenex) followed by streptavidin/peroxidase conjugate and developed with 3,3′-diaminobenzidine [11].

### Plasmids and transfection efficiency

Plasmids psCMVβgal (6.8 Kb) [11], psCMVtk (4.5 Kb) [11], psCMVcIFNβ (3.9 Kb) [7] and psCMVfIFNβ (3.9 Kb) carry respectively *Escherichia coli* β-galactosidase gene (3.5 Kb), herpes simplex thymidine kinase (1.2 Kb) and canine or feline IFNβ (0.6 Kb) in the polylinker site of psCMV (3.3 Kb), downstream of the CMV promoter and upstream of poly A sequences. The plasmids (bearing the kanamycin resistance gene for selection in *Escherichia coli*) were amplified, chromatographically purified and quality assessed as described [11]. Plasmid DNA for injection was resuspended to a final concentration of 2.0 mg/ml in sterile PBS.

### Liposome preparation and *in vitro* lipofection

DC-Chol (3β[N-(N′,N′-dimethylaminoethane)- carbamoyl cholesterol) was kindly supplied by BioSidus. DMRIE (1,2-dimyristyl oxypropyl-3-dimethyl- hydroxyethylammonium bromide) was kindly synthesized and provided by Dr. Eduardo M. Rustoy. DOPE (1,2-dioleoyl-sn-glycero-3-phosphatidyl ethanolamine) was purchased from Sigma. Liposomes were prepared at lipid/co-lipid molar ratios of 3:2 (DCChol:DOPE) or 1:1 (DMRIE:DOPE) by sonication as described [11]. Optimal lipid mixtures were determined for every cell line.

Cells, seeded onto 12-well plates at a density of 3-5x104 cells/cm2 were allowed to adhere overnight. Monolayers were exposed to lipoplexes (0.5 μg plasmid DNA/cm2 and 1 μl liposome/cm2) from 3 to 5 h in a serum- free medium. Then the lipofection medium was replaced with fresh complete medium. The feline and canine cells were lipofected with psCMV-fIFNβ and psCMV-cIFNβ respectively.

Lipofection rates were checked 24 h after lipofection by β-galactosidase staining with 5-bromo-4- chloro-3-indolyl β-D-galactopyranoside (X-GAL, Sigma) and further counting with an inverted phase contrast microscope [11].

### Sensitivity to bleomycin, suicide gene and IFNβ gene assays

Twenty-four hours after lipofection, with suicide gene (SG, HSV*tk*/GCV), feline/canine interferon-β (fIFNβ, cIFNβ) or βgal alone or co-delivered with 3 μg/ml bleomycin (Gador), cells were seeded on regular plates as monolayer (3.5 - 7.0 × 104 cells/ml) or on top of 1.5% solidified agar to form spheroids (1.0 x 105 cells/ml) and incubated with medium containing 5 µg/ml ganciclovir (Richet). After 5 days in monolayers or 12 days in spheroids, cell viability was quantified with acid phosphatase assay (APH) as described [7, 9].

### Flow cytometry cell cycle analysis and quantification of DNA fragmentation

Three days after lipofection, with SG, IFNβ, or βgal alone or co-delivered with BLM, cells were trypsinized, fixed in 70% (v/v) ethanol at -20°C for 1 h, treated with RNase, stained with 10 μg/ml propidium iodide (PI, Sigma) for 30 min, and subjected to single-channel flow cytometry on a Becton Dickinson FACScan (Franklin Lakes), with analysis of data performed using the Cylchred software (Cardiff University). Cells displaying a hypodipoid content of DNA indicative of DNA fragmentation were scored as apoptotic-necrotic.

### Quantification of necrotic cells

Three days after lipofection, with SG, IFNβ, or βgal alone or co-delivered with BLM, cells were harvested, freshly stained (without fixation) with PI solution (5 µg/ml) for 5 min, and then analyzed by flow cytometry as described above. PI enters the cells only if there is a loss of membrane integrity. Then, PI positive cells were scored as necrotic.

### Measurement of cellular reactive oxygen species (ROS) production

Three days after lipofection with SG, IFNβ, or βgal alone or co-delivered with BLM, cells were detached, washed with PBS and incubated with 0.5 µM H2DCF- DA (Invitrogen) in PBS [7]. After 20 min, normal culture conditions were re-established and the cellular fluorescence intensity was analyzed by flow cytometry. The final data were analyzed using the Flowing software (2.5.1, Finland) and the medium intensity of fluorescence was calculated (Geometric Mean: Gm).

### Determination of size and granularity or internal complexity of melanoma cells

Cell size and complexity of control untreated cells were analyzed by physical parameters of flow cytometry. Forward scatter intensity mainly correlates with cell area or size, and side scatter is a measure of the cell refractive index that depends on the cell granularity or internal complexity.

### Colony formation assay

Surviving cells after 3 days of BLM-gene treatments were trypsinized up to single cells. Monodispersed cells were seeded at low density and incubated at 37oC with complete medium until colonies were visible. To generate a similar number of colonies, it was necessary to seed different number of surviving cells: 500 (*Rn*), 1500 (*Dc*, *Bsk*) and 2500 (*Rk*) cells in a 6-well plate. Medium was changed once a week. After 7-10 days of culture, plates were washed, fixed with ethanol:acetic acid (3:1, v/v), and stained with crystal violet. The number of colonies was counted under an inverted microscope. The clonogenic capacity was defined as the percentage of seeded cells able to grow as colonies of more than 10 cells.

### Cloning efficiency in soft agar

Dispersed cells were resuspended in 0.25% agar in culture medium at 1x104 cells/ml and layered on top of 0.5% agar in culture medium. Three weeks after initiating cultures, colonies were counted.

### Melanosphere formation assay

Surviving cells after 3 days of BLM-gene treatments were trypsinized up to single cells and plated onto 12-well low attachment suspension culture plates (Greiner Bio- One) at a density of 2000 - 2500 viable cells/ml. Cells were grown in 1 ml serum-free media, supplemented with B27 (Gemini Bioproducts), and 20 ng/ml EGF. Melanospheres were counted after 6-8 days in culture. The melanosphere forming capacity was defined as the percentage of cells able of clonal proliferation as melanospheres with more than 8 cells.

### Statistics

Results were expressed as mean ± standard error of the mean (s.e.m.) (n: number of experiments corresponding to independent assays). Differences between groups were analyzed using unpaired Student's t-test (if two groups), one-way ANOVA followed by Tukey's test (if more than two groups) or two-way ANOVA followed by Bonferroni test (if two nominal variables). Correlations were determined by Pearson test with GraphPad Prism program (GraphPad Software Inc.).
